# The Impact of Histone Modifications in Endometriosis Highlights New Therapeutic Opportunities

**DOI:** 10.3390/cells12091227

**Published:** 2023-04-23

**Authors:** Iason Psilopatis, Kleio Vrettou, Florian Nima Fleckenstein, Stamatios Theocharis

**Affiliations:** 1 Department of Diagnostic and Interventional Radiology, Charité—Universitätsmedizin Berlin, Corporate Member of Freie Universität Berlin and Humboldt—Universität zu Berlin, Augustenburger Platz 1, 13353 Berlin, Germany; 2 First Department of Pathology, Medical School, National and Kapodistrian University of Athens, 75 Mikras Asias Street, Bld 10, Goudi, 11527 Athens, Greece; 3 BIH Charité Clinician Scientist Program, Berlin Institute of Health at Charité—Universitätsmedizin Berlin, BIH Biomedical Innovation Academy, 10117 Berlin, Germany

**Keywords:** histone, deacetylase, inhibitor, endometriosis, therapy

## Abstract

Endometriosis is a chronic disorder of the female reproductive system which afflicts a great number of women worldwide. Histone deacetylases (HDACs) prevent the relaxation of chromatin, thereby positively or negatively modulating gene transcription. The current review aims at studying the impact of histone modifications and their therapeutic targeting in endometriosis. In order to identify relevant studies, a literature review was conducted using the MEDLINE and LIVIVO databases. The current manuscript represents the most comprehensive, up-to-date review of the literature focusing on the particular role of HDACs and their inhibitors in the context of endometriosis. HDAC1, HDAC2, HDAC3, Sirtuin 1, and Sirtuin 3, are the five most studied HDAC enzymes which seem to, at least partly, influence the pathophysiology of endometriosis. Both well-established and novel HDACIs could possibly represent modern, efficacious anti-endometriotic drug agents. Altogether, histone modifications and their therapeutic targeting have been proven to have a strong impact on endometriosis.

## 1. Introduction

Endometriosis describes a chronic disease defined by the occurrence of endometrial tissue outside the uterus in women of reproductive age [1]. Clinical features of endometriosis vary depending on the location of the endometriotic lesions [2]. More accurately, even though affected individuals may remain asymptomatic, patients suffering from endometriosis usually claim chronic pelvic pain, which deteriorates before the onset of menstruation, infertility, vaginal bleeding or dyspareunia [3]. Other symptoms include dys-/hematuria, in the case of endometriotic lesions along the urinary tract, and dys-/hematochezia upon intestinal involvement [4]. Apart from a detailed patient history and a complete physical examination, the transvaginal ultrasound incorporates the best initial diagnostic test for endometriosis [5]. The uterus is generally not enlarged, whereas the presence of chocolate cysts or nodules in the bladder and/or the rectovaginal septum require further evaluation [6]. Laparoscopy shows endometriotic implants and adhesions and is, hence, regarded as the best available confirmatory test [7]. Expectant management is appropriate for most asymptomatic women [8]. For symptomatic endometriosis, empiric treatment with analgesics and continuous hormonal contraceptives is sufficient in cases of moderate pelvic pain and absence of complications [9]. Severe symptoms may require the administration of Gonadotropin-Releasing Hormone (GnRH) agonists or estrogen-progestin oral contraceptives [10,11]. In cases of non-response to pharmacological therapy or complicated disease courses, laparoscopic excision and ablation of endometrial implants represent the first-line surgical treatment method [12]. So far, the exact etiology of endometriosis is still not fully understood, with retrograde menstruation and coelomic metaplasia constituting the most prevalent pathogenetic hypotheses [13]. Other relevant theories include the hematogenous/lymphatic spread theory, the stem cell recruitment theory, as well as the embryogenetic theory [13]. 

**Figure 1:**
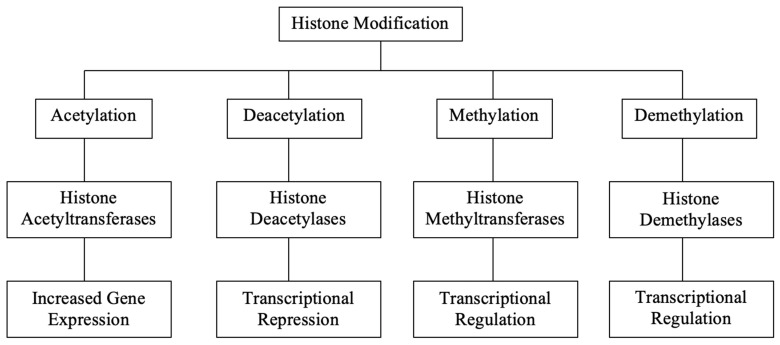
Schematic diagram of histone modifications.

In eukaryotic cell nuclei, DNA is tightly wrapped around a central histone octamer, hence forming the structure of the nucleosome to prevent gene transcription [14]. Histone modifications such as acetylation, deacetylation, methylation, and demethylation, permit the unwrapping of DNA and loosening of the nucleosome structure, in order to enable access of transcription factors to their target genes and initiate the transcriptional process (Figure 1) [15]. In this context, histone acetylation constitutes a dynamic process that involves the enzymes histone acetyltransferases (HATs) and histone deacetylases (HDACs) and modulates transcription by alternating the interaction of the negatively charged DNA strands with the positively charged histone proteins [16]. More precisely, HDACs remove acetyl groups from the histone lysine residues of histones and contribute to transcriptional repression in favor of chromatin compaction [16,17]. Based on their distinct structure and function, the eighteen, to date, identified HDACs may be categorized into four classes (class I-IV) and further subdivided into NAD^+^-dependent (class III) or Zn^2+^-dependent (class I, II and IV) enzymes [18,19]. The human Sirtuins (Sirtuin 1–7) are implicated in gene silencing, energy homeostasis, and mitochondrial function, with Sirtuin 1 primarily being a nuclear protein that targets the Peroxisome proliferator-activated receptor-Gamma Coactivator (PGC)-1alpha, Forkhead box protein O (FOXO), and Nuclear Factor-κB (NF-κB) families of transcription factors, among others [20].

In their critical capacity as epigenetic regulators, HDACs significantly influence the expression of various genes involved in numerous cellular processes ranging from cell proliferation or apoptosis, to cellular metabolism and immunogenicity [21]. Interestingly, HDAC inhibitors (HDACIs) represent novel therapeutic agents that may enhance histone acetylation by inhibiting the HDAC activity and, consequently, promote gene transcription [22]. 

Throughout the menstrual cycle, numerous global histone acetylation changes have been observed in the human endometrial tissue [23]. In detail, acetylation levels of H2AK5, H3K9, and H4K8, seem to be higher in the early proliferative phase, but subsequently tend to decline until ovulation. In the early secretory phase, H4K8 acetylation levels significantly rise and reach a peak during the mid-secretory phase. On the contrary, global histone acetylation levels in H2AK5, H3K9, and H4K8, experience a profound decline during the late secretory phase [24]. Except for their expression in the normal cyclic endometrium, HDACs seem to play an important role in endometrial pathologies, as well [25,26,27,28]. The present review of the literature aims at closely investigating the potential impact of histone modifications and their therapeutic targeting in endometriosis. 

## 2. The Role of HDACs and Sirtuins in the Pathogenesis of Endometriosis 

A great number of studies have, to date, incorporated different endometriotic cell lines, animal models, as well as clinical tissue samples, in order to examine the role of HDACs in the pathogenesis of endometriosis.

To begin with, Xiaomeng et al. monitored the histone modification profile of patients suffering from endometriosis and distinguished low histone H4 acetylation levels in both eutopic and ectopic endometrial tissues. Moreover, Sirtuin 1 mRNA levels showed significant downregulation in eutopic endometrium, HDAC1 mRNA levels were significantly decreased in ectopic endometrium, whereas HDAC2 mRNA levels were, in contrast, significantly upregulated in eutopic endometrial tissue [29]. On the contrary, Samartzis et al. studied the expression of HDACs in 74 endometriosis tissue samples and concluded that HDAC1, but not HDAC2 or HDAC3, is significantly overexpressed in endometriosis and correlates with the expression of estrogen and progesterone hormone receptors [30]. Furthermore, Zhang et al. explored the molecular mechanism of the exosomal long non-coding RNA Homeobox Transcript Antisense RNA (HOTAIR) in the progression of endometriosis, which was shown to augment HDAC1 expression [31]. Colón-Díaz et al. performed Western blot analysis in endometriotic versus endometrial stromal cells and described significantly elevated basal *HDAC1/2* expression levels in endometriosis. Following the application of estradiol and progesterone in the Hs832cT endometriotic cell line, *HDAC1/2* expression did not, nonetheless, experience significant alterations. In patients suffering from endometriosis, immunohistochemical analysis confirmed a substantial expression of HDAC1/2 proteins, which varied depending on lesion localization. More precisely, HDAC1 was mostly expressed in dermal, ovarian, and gastrointestinal lesions, whereas the expression of HDAC2 seemed to be stronger in skin lesions and the endometrial tissue [32]. Moreover, Mai et al. observed low Hepatocyte Nuclear Factor 4A (HNF4A) expression, in accordance with HDAC2 upregulation, in both the endometriosis cell line hEM15A and patient-derived endometriosis tissue samples. In hEM15A cells, HDAC2 silencing correlated with reduced cell viability and invasion, as well as apoptosis induction. HDAC2 silencing in the clinical tissue samples and in vivo accordingly resulted in smaller lesions, downregulated cell proliferation and enhanced apoptosis [33]. Veena et al. studied the involvement of HDACs in the risk of endometriosis for women of South Indian origin and described that the polymorphism of *HDAC1* rs1741981 seems to significantly increase the risk of endometriosis in the South Indian population. On the contrary, single-nucleotide polymorphisms of *Sirtuin 1* and *Sirtuin 3* did not correlate with the disease in South Indian patients [34]. In 2019, Kim et al. first identified low HDAC3 protein levels in eutopic endometrium of infertile patients with endometriosis and, consecutively, induced endometriosis in primate and mouse models, which resulted in the diminution of HDAC3 expression, as well as in implantation and decidualization deficiencies [35]. Three years later, the same study group inspected the role of Sirtuin 1 in endometriosis and observed significant overexpression in both epithelial and stromal cells of patients with endometriosis. Interestingly, high Sirtuin 1 levels in endometriotic lesions seemed to cause further disease aggravation, whereas the Sirtuin 1 inhibitor EX-527 restored implantation and inhibited the development of endometriosis in mice [36]. Wang et al. stated that endometrial epithelial cells show significant Sirtuin 1 upregulation in endometriotic human cell lines or clinical specimens, thereby prompting the epithelial-mesenchymal transition by evading damage or senescence during disease progression [37]. Additionally, Mvunta et al. revealed that Sirtuin 1 expression seems to be higher in endometriosis with ovarian carcinoma than in normal ovaries [38]. Similarly, Teasley et al. reported increased nuclear Sirtuin 1 expression in human endometrial samples from regularly cycling women with endometriosis and endometriosis-associated ovarian cancer patients, which even correlated with Kirsten rat sarcoma virus (KRAS) expression in the latter group [39]. Yoo et al. suggested that Sirtuin 1 upregulation in the eutopic endometrium of patients suffering from endometriosis was positively correlated with the expression of B-Cell Lymphoma 6 (BCL6) throughout the menstrual cycle phases. This aberrant Sirtuin 1 activation was further confirmed in a primate model of endometriosis progression [40]. By performing Enzyme-Linked Immunoassay (ELISA) in serum, plasma, urine, and cervical mucus samples from patients suffering from endometriosis, Sansone et al. measured significantly higher Sirtuin 1 levels in sera of women with advanced stage endometriosis [41]. Taguchi et al. obtained stromal cells from human ovarian endometriomas and demonstrated Sirtuin 1 expression in both endometriotic and normal endometrial stromal cells. In endometriotic stromal cells, the Sirtuin activator resveratrol blocked Tumor Necrosis Factor (TNF)-α-mediated interleukin (IL)-8 release in a dose-dependent manner, whereas the Sirtuin inhibitor sirtinol boosted IL-8 release [42]. González-Fernández et al. employed quantitative Reverse Transcription—Polymerase Chain Reaction (qRT-PCR) to examine *Sirtuin* expression in human mural granulosa-lutein cells from endometriosis patients and demonstrated that Follicle-Stimulating Hormone (FSH) and Luteinizing Hormone (LH) administration doses are positively associated with *Sirtuin 1*, *Sirtuin 6*, and *Sirtuin 7*, levels in in vitro fertilization patients [43]. Lastly, Kaleler et al. collected serum and ovarian endometrioma tissue samples from a total of thirty women with endometriosis and found Sirtuin 3 levels to show significant downregulation in endometrioma tissue samples, yet no statistically significant differences in patient versus control serum samples. In accordance with these observations, Glutamate Dehydrogenase (GDH), Succinate Dehydrogenase (SDH), and Manganese Superoxide Dismutase (MnSOD) all target enzymes of Sirtuin 3 [44], and showed decreased enzyme activities in endometrioma tissue homogenates, as well as in mitochondria (except for MnSOD, which had an increased activity in mitochondria) [45].

Table 1 briefly summarizes the aforementioned study results.

Altogether, these results indicate that diverse HDACs seem to be involved in the pathogenesis of endometriosis and may even act as biomarkers for disease diagnosis and/or progression. Nevertheless, different research groups came to (partly) contradictory conclusions in terms of HDAC expression levels in eutopic and ectopic endometrium.

## 3. HDACIs as Potential Novel Agents for Endometriosis Therapy

### 3.1. Introduction to HDACIs

Table 2 provides a brief overview of the standard HDACIs that seem to represent promising novel agents for endometriosis therapy.

### 3.2. Multiple HDACIs

Several study groups have applied different HDACIs to endometriotic cells with a view to determining their therapeutic potential.

Chadchan et al. first treated immortalized Human Endometriotic Epithelial Cells/Luciferase (iHEECs/Luc) with n-butyrate, trichostatin-A, suberoylanilide hydroxamic acid, or entinostat, which all elevated histone H3 acetylation levels and inhibited cell viability [46]. Staining of ectopic endometriotic lesions of mice with anti-HDAC1 antibodies revealed an abundant HDAC1 expression, while intraperitoneal injections of n-butyrate, trichostatin-A, suberoylanilide hydroxamic acid, or entinostat, in this model resulted in smaller and fewer endometriotic lesions with a thinner stroma and epithelium [46]. In 2011, Kawano et al., after demonstrating that the levels of acetylated histones in the endometriotic cyst stromal cells are significantly lower than in normal endometrial stromal cells, treated the endometriotic cyst stromal cells with valproic acid, suberoylanilide hydroxamic acid, and apicidin. This pharmacological HDAC inhibition not only led to cell proliferation repression and cell cycle arrest or apoptosis induction, but also promoted histone H3/H4 acetylation in the promoter regions of numerous genes such as *cycle checkpoint kinase 2*, *p16^INK4a^*, *p21^Waf1/Cip1^*, and *p27^Kip1^*, thus inducing the expression of these cell cycle regulatory proteins, as well as diminishing the B-cell lymphoma 2 (Bcl-2) and B-cell lymphoma-extra-large (Bcl-xL) protein expression [47]. Two years later, the same research group focused on the effect of valproic acid on endometriotic cyst stromal cells, which was found to promote the acetylation of histones H3 and H4 in the *CCAAT Enhancer Binding Protein α* (*C/EBPα*) promoter region and, as a result, to increase the respective mRNA and protein expression levels [48].

### 3.3. Valproic Acid

Valproic acid is a short chain fatty acid that has long been approved for the treatment of epileptic patients [49]. 

Kai et al. isolated endometriotic cyst stromal cells from ovarian endometriotic tissues with a view to investigating the effect of valproic acid on the expression of Death Receptor 6 (DR6) in endometriosis [50]. The expression of DR6 was significantly boosted in both endometriotic cyst stromal cells and ovarian endometriotic tissues after valproic acid stimulation, alongside a profound acetylation of histone H4 in the *DR6* gene promoter region [50]. Furthermore, Liu et al. induced endometriosis in 77 adult female rats, which were then treated with valproic acid and/or progesterone. Combinational agent administration resulted in significantly smaller lesions, better response to deleterious thermal stimuli, as well as an increase in weight gain [51]. Zhao et al. applied a combinational treatment of levo-tetrahydropalmatine and valproic acid to a rat model of endometriosis, which resulted in significantly smaller endometriotic lesions, a better response to noxious thermal stimuli, as well as a reduced immunoreactivity to HDAC2 in dorsal root ganglia. Notably, generalized hyperalgesia was further alleviated through central desensitization, alongside a decreased response to tyrosine kinase receptor A and/or calcitonin gene-related peptide in eutopic and ectopic endometrial tissue [52].

### 3.4. Suberoylanilide Hydroxamic Acid

Vorinostat, or suberoylanilide hydroxamic acid, is a standard pan-HDACI that interacts with and blocks the catalytic site of all HDAC classes [53,54]. 

Kim et al. treated eutopic and ectopic human endometrial stromal cells with suberoylanilide hydroxamic acid and concluded that the aforementioned HDACI significantly upregulated the Thioredoxin (TRX) Binding Protein-2 (TBP-2) mRNA and protein expression, led to the reduction of the TRX/TBP-2 ratio, induced apoptosis, and halted the transfection of short-interfering TRX [55]. Moreover, Zheng et al. first induced endometriosis in Krüppel-like factor 11 (Klf11) -/- mice through surgical autologous uterine tissue implantation and treated them with suberoylanilide hydroxamic acid, but did not observe any changes in collagen 1A1 expression and, consequently, the extent of fibrosis. Subsequent endometriosis induction in wild-type mice, followed by the administration of suberoylanilide hydroxamic acid, however, led to collagen 1A1 activation (mediated by the hyperacetylation of the relevant promoter) and significant fibrotic disease progression [56]. Overall, the results of the present relevant original research studies seem to be contradictory, concerning the role of vorinostat in the treatment of endometriosis.

### 3.5. Trichostatin A

Trichostatin A, an antifungal antibiotic initially isolated from Streptomyces hygroscopicus, constitutes an effective and precise hydroxamic acid [57]. 

Wu et al. intensively focused on the impact of trichostatin A on endometriosis and published a total of three relevant original research articles. In 2007, the group first discovered that trichostatin A may not only lessen the invasiveness but also reactivate the expression of E-cadherin in the 11Z and 22B endometriotic cell lines [58]. A year later, Wu et al. again cultured the same endometriotic cell lines with trichostatin A and reported an elevated Peroxisome Proliferator-Activating Receptor γ (PPARγ) expression in a dose-dependent manner [59]. In their third published work on this topic, they outlined the trichostatin-A-mediated attenuation of NF-κB activation, alongside the suppression of p65 phosphorylation and nuclear translocation in the aforementioned endometriotic cell lines [60]. Seo et al. treated human endometriotic stromal cells with trichostatin A, which reduced cell viability and induced both apoptosis and nonsteroidal anti-inflammatory drug-activated gene-1 mRNA and protein expression in a dose-dependent way [61]. Moreover, Lu et al. induced endometriosis in 30 adult female rats, which were then treated with trichostatin A. The aforementioned HDACI caused a significant reduction in endometriotic lesion size, a more adequate response to nocifensive stimuli, as well as a lower immunoreactivity to Transient Receptor Potential Vanilloid 1 (TRPV1) in eutopic endometrial tissue, to Protein Kinase C-like 1 (PKC1) in ectopic endometrium and to Protein Gene Product 9.5 (PGP 9.5) in the vagina [62]. Taken altogether, trichostatin A seems to represent a potent HDACI for the treatment of endometriosis. 

### 3.6. Romidepsin

Romidepsin represents a structurally unique bicyclic HDACI that selectively inhibits class I HDACs [63].

By immortalizing primary peritoneal epithelial endometriotic cells, Imesch et al. generated an appropriate in vitro model in order to study the epigenetic effects of the HDACI romidepsin on endometriosis. Romidepsin was found to promote histone protein acetylation via HDAC inhibition, to upregulate p21, and to have a negative impact on cyclins B1 and D1. As a consequence, romidepsin seemingly succeeds in reducing proliferation and activating apoptotic cell death in 11z immortalized epithelial endometriotic cells [64]. In 2011, the same study group published their second original research article on the potential role of romidepsin in the treatment of endometriosis. This time, romidepsin treatment repressed both *Vascular Endothelial Growth Factor* (*VEGF*) gene transcription and VEGF protein expression in 11z human endometriotic cells. Furthermore, VEGF protein secretion into the culture medium was also blocked, whereas the Hypoxia-Inducible Factor-1α (HIF-1α) expression was downregulated in cobalt chloride-pretreated endometriotic cell cultures [65]. Two years later, the researchers decided to treat the immortalized epithelial endometriotic cells with romidepsin, suberoylanilide hydroxamic acid, or the G-Protein-coupled Estrogen Receptor 1 (GPER1) antagonist G-15. Both romidepsin and suberoylanilide hydroxamic acid provoked an accumulation of acetylated histones in a concentration-dependent manner, thereby decreasing GPER1 expression. Notably, the pre-administration of the GPER1 agonist G-1 to endometriotic cells induced rapid Akt phosphorylation and stimulated cell proliferation, whereas G-15 accounted for the exact opposite effects [66].

### 3.7. Panobinostat

Panobinostat is another HDACI that has received Food and Drug Administration (FDA) approval for the treatment of multiple myeloma [67]. 

No original research article has, to date, been published on the potential role of panobinostat in the treatment of endometriosis.

### 3.8. Alternative HDACIs

Certain study groups have also explored the role of diverse alternative/natural therapeutic agents as efficient HDACIs for the treatment of endometriosis. 

Correa et al. administered the natural product from the Garcinia Indica fruit garcinol, in combination with a Transforming Growth Factor beta (TGF-β) type 1 receptor inhibitor (TGFβR1I), to wild-type control and Klf11 -/- mice and proved that the two pharmacological agents synergistically regulated Klf11-mediated transcription, as well as inhibited endometriosis progression and lesional human cytochrome P450 3A (CYP3A) expression [68]. Furthermore, Eisalou et al. assessed the Western blotting expression of Sirtuin 1 in rat models with surgically induced endometriotic lesions and showed that Sirtuin 1 protein levels were significantly higher in rats treated with a selected dose of 6000 μg/kg of gamma-oryzanol, a ferulic acid ester of sterols extracted from rice bran oil, for the duration of one month [69]. Li et al. stated that the traditional Chinese therapeutic agent Bushen Wenyang Huayu Decoction (BWHD) may downregulate autophagy through Sirtuin 1 upregulation in endometriosis rats [70]. Last but not least, Zhou et al. employed an in vitro endometriosis study model and were able to prove high Sirtuin 1 levels after ferric ammonium citrate treatment in eutopic endometrial stromal cells. Interestingly, silencing of Sirtuin 1 reversed iron overload-mediated Poly (ADP-ribose)-Polymerase 1 (PARP1) deactivation and autophagy stimulation [71].

Table 3 comprehensively presents the effects of the studied HDACIs for the treatment of endometriosis.

## 4. Discussion and Conclusions

Posttranslational modifications of histones may modulate gene transcription, chromatin remodeling, as well as the architecture of the cellular nucleus; hence, representing one of the hallmarks of cancer [72]. More precisely, HDACs may even deacetylate a wide array of non-histone cellular substrates, that regulate various biological mechanisms responsible for cancer development and progression [72]. In this context, HDACIs facilitate the hyperacetylation of (non-)histone targets, thereby enabling the restoration of cellular acetylation homeostasis and reestablishing the appropriate expression and function of diverse proteins that could help counteract carcinogenesis [72]. Apart from their crucial role in the pathophysiology of cancer initiation and progression, epigenetic histone modifications, alongside their therapeutic targeting, have a profound impact on non-malignant health conditions, respectively [73]. In cardiovascular diseases, HDACs seem to be closely related to the pathogenesis of atherosclerosis, arrhythmia, myocardial infarction, cardiac hypertrophy, cardiac fibrosis, as well as vascular calcification [73]. Moreover, HDACIs have exhibited great therapeutic potential in neurodegenerative diseases, inflammatory conditions, and osteoporosis [73]. 

In the field of gynecology and obstetrics, HDACIs have long been proposed as potent alternative drug agents for the treatment of diverse gynecological cancer entities including uterine, cervical, ovarian, and breast cancer [26,74,75,76]. As far as benign gynecological health conditions are concerned, Olaniyi et al. have, for instance, suggested that the short chain fatty acid acetate successfully restores the ovarian function in experimentally induced polycystic ovarian syndrome (PCOS) rat models [77]. In addition, Sunita et al. concluded that HDACIs embody effective therapeutic agents against infertility through the efficient restoration of normal reproductive mechanisms [78]. The present review of the literature, to our knowledge, constitutes the most comprehensive, up-to-date review article on the impact of histone modifications and their therapeutic targeting in endometriosis, a benign gynecological health disease which negatively affects the quality of life of many women worldwide. 

HDAC1, HDAC2, HDAC3, Sirtuin 1, as well as Sirtuin 3, are the five most studied HDAC enzymes that seem to play an important role in the pathophysiology of endometriosis. Specifically, most study groups have placed special focus on the expression of the class I HDAC HDAC1 and the class III HDAC Sirtuin 1, but reached partly controversial conclusions concerning the up- or downregulation of these two enzymes in eutopic and/or ectopic endometrium of women suffering from endometriosis. The example of endometriosis is not the only case in which different research groups have made opposing observations regarding the expression of specific molecules [79,80,81,82,83,84,85]. Larger collectives (cell lines, animal models, tissue samples) need, therefore, to be employed in order to reduce bias and ensure the extraction of reproducible study results. In addition, as there are currently 18 enzymes, future research should focus more on HDACs other than HDAC1, HDAC2, HDAC3, Sirtuin 1, or Sirtuin 3, with a view to ideally discovering the potential role of the remaining members of the HDAC family in endometriosis.

Valproic acid, suberoylanilide hydroxamic acid, trichostatin A, as well as romidepsin, are the four standard HDACIs that, alone or in combination with other drug agents, exhibit a promising therapeutic effect in endometriosis by inhibiting disease progression (or even enhancing its regression), counteracting cellular and/or immune dysregulations, as well as having a positive effect on pain stimulation and transmission. Furthermore, except for the aforementioned standard HDACIs, phytopharmaceuticals have been also reported to act as potent HDACIs in endometriosis, thereby highlighting the usefulness of natural products and reviving the hope for women who suffer from endometriosis, but do not wish to receive standard pharmacotherapy. Even though these remarks might seem groundbreaking, no study group has, so far, examined the effects of HDACIs in the context of randomized clinical trials incorporating healthy women and patients with endometriosis. The conceptualization of such trials is of utmost significance in order to verify the clinical utility and safety of diverse HDACIs in the treatment of endometriosis patients and to explore eventual adverse side effects following their planned administration to women. Given that expression of HDACs is ubiquitous and can be found in nearly all tissues, a local therapy in the pelvis via an intra-uterine device coated with HDACIs would eventually represent, if feasible, a safer option than systemic application [86].

In summary, histone modifications and their therapeutic targeting were demonstrated to have a strong impact in endometriosis. Future research should, therefore, focus more on the role of epigenetic alterations, with a view to gaining better insights into this dark box called ‘endometriosis’.

## Figures and Tables

**Table 1:** The role of different HDACs in the pathogenesis of endometriosis.

HDAC	Role in Endometriosis	References
HDAC1	Significantly higher basal HDAC1 gene expression levels in endometriotic versus endometrial stromal cells	[32]
	Significantly downregulated HDAC1 mRNA expression levels in ectopic endometrium	[29]
	Significantly elevated immunoreactivity score levels of HDAC1 in endometriosis	[30]
	Positive correlation of HDAC1 with the expression of estrogen receptor-α and estrogen receptor-β Negative correlation of HDAC1 with the expression of the epithelial progesterone expression	[30]
	Significant HDAC1 protein overexpression in dermal, ovarian, and gastrointestinal lesions	[32]
	rs1741981 polymorphism significantly exponentiates the risk of endometriosis in the South Indian population	[34]
HDAC2	Significantly higher basal HDAC2 gene expression levels in endometriotic versus endometrial stromal cells	[32]
	Significant HDAC2 mRNA upregulation in eutopic endometrium	[29]
	Significantly higher HDAC2 protein levels in endometriosis tissues	[33]
	Significant HDAC2 protein overexpression in skin lesions and endometrium from patients with endometriosis	[32]
HDAC3	Low HDAC3 protein levels in eutopic endometrium of infertile women with endometriosis	[35]
Sirtuin 1	Significantly decreased Sirtuin 1 mRNA expression levels in eutopic endometrium	[29]
	Significant Sirtuin 1 protein overexpression in both epithelial and stromal cells of endometriosis patients	[36,40]
	Significantly higher Sirtuin 1 protein levels in sera of women with advanced stage endometriosis	[41]
	Further disease aggravation	[36]
	Promotion of epithelial-mesenchymal transition during disease progression	[37]
	More profound in endometriosis with ovarian carcinoma Correlation with KRAS expression	[38,39]
	Positive correlation with the expression of BCL6 throughout the menstrual cycle phases	[40]
Sirtuin 3	Significantly decreased Sirtuin 3 protein expression levels in endometrioma tissue samples	[45]

**Table 2:** Overview of the studied standard HDACIs.

Class	HDACI	Target HDAC
Hydroxamic acids	Trichostatin A Suberoylanilide hydroxamic acid	Pan Pan
Short-chain fatty acids	N-butyrate Valproic Acid	I, II I, IIa
Benzamides	Entinostat	I
Cyclic peptides	Apicidin Romidepsin	I I

**Table 3:** The therapeutic role of HDACIs as drug agents for endometriosis.

HDACI	Role in Endometriosis	References
N-butyrate	Histone H3 hyperacetylation Cell viability inhibition Smaller and fewer endometriotic lesions with a thinner stroma and epithelium	[46]
Entinostat	Histone H3 hyperacetylation Cell viability inhibition Smaller and fewer endometriotic lesions with a thinner stroma and epithelium	[46]
Apicidin	Cell proliferation repression Cell cycle arrest Apoptosis induction Histone H3/H4 hyperacetylation in the promoter regions of *cycle checkpoint kinase 2*, *p16^INK4a^*, *p21^Waf1/Cip1^*, and *p27^Kip1^* Reduced Bcl-2 and Bcl-xL protein expression	[47]
Valproic acid	Cell proliferation repression Cell cycle arrest Apoptosis induction Histone H3/H4 hyperacetylation in the promoter regions of *cycle checkpoint kinase 2*, *C/EBPα*, *p16^INK4a^*, *p21^Waf1/Cip1^*, and *p27^Kip1^* Reduced Bcl-2 and Bcl-xL protein expression Enhanced DR6 expression Significantly smaller lesions, better response to deleterious thermal stimuli, and increase in weight gain after combination with progesterone Significantly smaller lesions, better response to noxious thermal stimuli, reduced immunoreactivity to HDAC2 in dorsal root ganglia, as well as generalized hyperalgesia alleviation after combinational treatment with levo-tetrahydropalmatine	[47,48,50,51,52]
Vorinostat	Cell viability inhibition Smaller and fewer lesions with a thinner stroma and epithelium Cell cycle arrest Apoptosis induction Histone H3/H4 hyperacetylation in the promoter regions of *cycle checkpoint kinase 2*, *p16^INK4a^*, *p21^Waf1/Cip1^*, and *p27^Kip1^* Reduced Bcl-2 and Bcl-xL protein expression Significant TBP-2 upregulation Reduction of the TRX/TBP-2 ratio Collagen 1A1 activation Significant fibrotic disease progression Decreased GPER1 expression	[46,47,55,56]
Trichostatin A	Histone H3 hyperacetylation Cell viability inhibition Smaller and fewer lesions with a thinner stroma and epithelium Less invasiveness E-cadherin reactivation Elevated PPARγ expression NF-κB attenuation Suppression of p65 phosphorylation and nuclear translocation More adequate response to nocifensive stimuli Induced nonsteroidal anti-inflammatory drug-activated gene-1 mRNA and protein expression Lower immunoreactivity to TRPV1 in eutopic endometrial tissue, to PKC1 in ectopic endometrium, and to PGP 9.5 in the vagina	[46,58,59,60,61,62]
Romidepsin	p21 upregulation Low cyclin B1 and D1 levels Reduced cell proliferation Apoptotic cell death activation VEGF repression HIF-1α downregulation in cobalt chloride-pretreated cells Decreased GPER1 expression	[64,65,66]
Garcinol	Regulation of Klf11-mediated transcription, reduced endometriosis progression and low lesional human CYP3A expression after combination with TGFβR1I	[68]
Gamma-oryzanol	Elevated Sirtuin 1 protein levels	[69]
BWHD	Autophagy downregulation through Sirtuin 1 upregulation	[70]
Ferric ammonium citrate	Elevated Sirtuin 1 levels	[71]

## Data Availability

Not applicable.
